# Fructosamine and glycated hemoglobin as biomarkers of glycemic control in people with type 2 diabetes mellitus and cancer (GlicoOnco study)

**DOI:** 10.1016/j.clinsp.2023.100240

**Published:** 2023-06-28

**Authors:** Marcos Tadashi Kakitani Toyoshima, Priscilla Cukier, Aline Santos Damascena, Rafael Loch Batista, Fernanda de Azevedo Correa, Eduardo Zanatta Kawahara, Carlos André Minanni, Ana O. Hoff, Marcia Nery

**Affiliations:** a Serviço de Onco-Endocrinologia, Instituto do Câncer do Estado de São Paulo Octávio Frias de Oliveira; Hospital das Clínicas da Faculdade de Medicina da Universidade de São Paulo, São Paulo, SP, Brazil; b Serviço de Endocrinologia e Metabologia. Hospital das Clínicas da Faculdade de Medicina da Universidade de São Paulo, São Paulo, SP, Brazil; c Departamento de Patologia, Faculdade de Medicina da Universidade de São Paulo, São Paulo, SP, Brazil; d Hospital Israelita Albert Einstein, São Paulo, SP, Brazil

**Keywords:** Chemotherapy, Glucocorticoids, Cancer, Glycemic control, Fructosamine, HbA1c

## Abstract

**Introduction:**

Glycemic control is important to avoid diabetes complications in individuals with cancer. There is no evidence for HbA1c and fructosamine as reliable biomarkers in these conditions. There are particularities in caring for patients with diabetes and cancer that can alter these biomarkers.

**Objective:**

The aim of this study was to evaluate HbA1c and fructosamine as glycemic biomarkers in people with type 2 diabetes and cancer, undergoing clinical or surgical oncological treatment.

**Methods:**

The authors conducted a single-center, retrospective analysis with people who have cancer and diabetes. Comparison of glycemic biomarkers (HbA1c, fructosamine, and Self-Monitoring of Blood Glucose [SMBG]) was performed including evaluation in individuals undergoing chemotherapy, using glucocorticoids, with anemia, hypoproteinemia or with reduced estimated Glomerular Filtration Rate (eGFR).

**Results:**

There was a strong positive correlation between fructosamine and HbA1c (n = 318, *r* = 0.66, p < 0.001) in people with diabetes and cancer even in those under chemotherapy (n = 101, *r* = 0.61, p < 0.001) or using glucocorticoids (n = 96, *r* = 0.67, p<0.001). There was a strong correlation between HbA1c and fructosamine in subjects with anemia (n = 111, *r* = 0.66, p < 0.001), hypoproteinemia (n = 54, *r* = 0.67, p < 0.001), or with eGFR ≥ 60 mL/min/1.73 m^2^ (n = 189, *r* = 0.70, p < 0.001), and moderate correlation with hypoalbuminemia (n = 21, *r* = 0.54, p = 0.001) and with reduced eGFR (n = 67, *r* = 0.57, p < 0.001). The correlations between fructosamine and HbA1c with SMBG were moderate (n = 164, *r* = 0.49, p < 0.001; n = 111, *r* = 0.55, p < 0.001, respectively), strong in subjects undergoing chemotherapy, with hypoalbuminemia or hypoproteinemia, and at least moderate, if eGFR < 60 mL/min/1.73 m^2^ or with anemia.

**Conclusions:**

Fructosamine and HbA1c can be used as glycemic biomarkers in people with diabetes and cancer, even in those with anemia, hypoproteinemia, or undergoing chemotherapy.

## Highlights

• Fructosamine and HbA1c are associated in individuals with diabetes and cancer, even on chemotherapy.
• HbA1c and fructosamine are associated with self-monitoring of blood glucose in cancer.
• The association between fructosamine and HbA1c occurs even in anemia and hypoproteinemia.

## Introduction

Cancer and diabetes are leading causes of morbidity and mortality worldwide.^1^ The coexistence of cancer and diabetes mellitus has been increasingly studied, especially the association between diabetes and cancer risk.^1^^,^^2^ The prevalence of diabetes in cancer subjects reaches 30%.^3^ However, there is a dearth of scientific studies describing diabetes control in oncology practice.^4^

Glycemic control is extremely important for the prevention of acute and chronic complications of diabetes. Traditionally, glycated Hemoglobin (HbA1c) has been used as the standard measure for long-term glucose control and as a metric in clinical trials to predict complications associated with diabetes.^5^^,^^6^ Continuous Glucose Monitoring (CGM) and Self-Monitoring of Blood Glucose (SMBG) are other available tools for health providers and subjects to assess the effectiveness of the management plan on glycemic control.^5^ Serum fructosamine has been proposed useful tool for monitoring short-term glycemic control^7^, ^8^, ^9^, ^10^, including cancer subjects.^3^ However, fructosamine is still underutilized in clinical practice and in clinical studies.^7^^,^^9^ Furthermore, fructosamine has been shown to be an independent biomarker for predicting microvascular complications of diabetes, such as diabetic retinopathy and nephropathy.^9^

There are some particularities in the management of subjects with diabetes and cancer, besides several questions to be answered in this regard. Can HbA1c and fructosamine be used as glycemic biomarkers in cancer patients undergoing cancer treatment?^11^ Considering the high incidence of anemia in these subjects, is it possible to trust the HbA1c test?^3^ Cancer and chemotherapy are causes of cachexia and malnutrition. Hypoproteinemia, especially hypoalbuminemia, is a marker of cancer-associated cachexia and malnutrition.^12^ Can cancer subjects and hypoproteinemia or hypoalbuminemia be evaluated with serum fructosamine?

Due to doubts about the use of HbA1c and fructosamine as methods of glycemic monitoring in individuals with cancer and diabetes, the authors used SMBG data as the standard method. However, capillary blood glucose strips are not freely available to all individuals in Brazil through the Unified Health System (SUS ‒ Sistema Único de Saúde) and the cost of blood glucose monitoring is still high. Laboratory assays for serum fructosamine are inexpensive, easy to perform^13^, and could be an alternative to SMBG to detect acute glycemic changes in a patient with diabetes and cancer, especially individuals undergoing cancer treatment.

In order to answer those questions, the aim was to evaluate the HbA1c and fructosamine as glycemic biomarkers in patients with type 2 diabetes and cancer, undergoing chemotherapy and with common clinical settings that may interfere with them, such as anemia and hypoproteinemia.

## Material and methods

### Study design

A retrospective study was carried out of all eligible subjects with cancer and type 2 diabetes and aged 18 years or older referred to the Onco-Endocrinology outpatient clinic at Instituto do Cancer do Estado de São Paulo, Hospital das Clínicas, University of Sao Paulo School of Medicine, and who had data related to glycemic control between June 2014 and July 2022. This outpatient clinic exclusively follows patients with diabetes undergoing medical or surgical treatment for cancer, not following patients with cancer without active cancer treatment. Subjects on peritoneal dialysis or hemodialysis, type 1 diabetes, and those with Chronic Kidney Disease (CKD) with estimated Glomerular Filtration Rate (eGFR) < 15 mL/min/1.73 m^2^ or recent blood transfusion were not included in the analysis. Data from individuals with hematologic malignancies involving paraproteins were excluded from analyzes involving fructosamine. Three independent groups of analyzes were used to study the association between HbA1c and fructosamine, fructosamine and 1-month SMBG, HbA1c, and 3-month SMBG. Applying the non-inclusion criteria, the number of subjects studied in each of them was: 318 in HbA1c vs. fructosamine study, 164 in the fructosamine vs. 1-month SMBG study, and 111 in HbA1c and 3-month SMBG study. The study protocol was approved by the local ethics review committee (CAAE 51230915.8.0000.0065). The reporting of this study conforms to STROBE guidelines.^14^

Medical records were analyzed to collect data on demographics, medical history, need for hemodialysis, use of glucocorticoids, and chemotherapy. Outpatient laboratory values of serum fructosamine, HbA1c, total protein, albumin, and hemoglobin were evaluated, as well as self-management blood glucose (SMBG) records. The recommendation for performing SMBG was at least two to three measurements of capillary blood glucose per day before meals, and postprandial measurements are requested by the clinical decision of the attending physician. For the analysis of this study, SMBG data were considered arbitrarily valid if there were at least 30 measurements per month. The eGFR was calculated using the CKD-EPI equation^15^ and abnormal renal function was defined as eGFR < 60 mL/min/1.73 m^2^. Fructosamine and HbA1c are routinely measured every three months on the same sample. HbA1c was determined by high-performance liquid chromatography, certified by the National Glyco Hemoglobin Standardization Program (NGSP-USA). Fructosamine dosage was determined by an automated colorimetric enzymatic method (Labtest do Brasil, Minas Gerais, Brazil). Anemia was defined as a hemoglobin level < 12 g/dL in both sexes.^16^ Hypoalbuminemia was defined in the present study as an albumin level ≤ 3.5 mg/dL and hypoproteinemia was defined as a serum total protein level ≤ 6.5 mg/dL^17^, whose cutoffs are defined by the laboratory.

### Statistical analysis

It was estimated that the sample of 85 subjects was sufficient to estimate correlations greater than 0.3, considering a significance level of 0.05 with a power of 0.8.^18^

Due to the presence of missing data mainly related to SMBG, three different datasets were analyzed with data available in each of them: HbA1c vs. fructosamine, fructosamine vs. 1-month SMBG, and HbA1c vs. 3-month SMBG.

Descriptive statistics were performed, with data presented in absolute (n) and relative frequency (%), mean (standard deviation), or median (first quartile – third quartile). Pearson's correlation coefficient with a 95% Confidence Interval was performed to measure the strength of a linear association between two variables. For the correlation between fructosamine and SMBG, the mean SMBG of the last month of the fructosamine test was used; and for the correlation between HbA1c and SMBG, the mean of the SMBG measurements over the last three months of the HbA1c exam. Correlation tests were also performed according to the anemia status, in the analysis involving HbA1c, and with serum albumin and with total serum protein status, in the analysis involving fructosamine. The correlation values obtained were classified according to Dancey and Reidy:^19^ 0.10< *r* <0.40 weak correlation, 0.40≤ *r* <0.60 moderate correlation, and 0.60≤ *r* ≤1 strong correlation. The significance level adopted was 5% and the free statistical software R version 4.0.2 (www.r-project.org) was used in the analyses.

## Results

Table 1 summarizes the baseline characteristics of the subjects with diabetes mellitus and cancer included in each of the three main study analyses. Applying the non-inclusion criteria and considering all three studies, 318 individuals were included. Of these, 90 subjects were analyzed simultaneously in the three studies. The median age in the population of the three study analyses was around 65 years. The most frequent sites of cancers in HbA1c vs. fructosamine analysis were breast, colorectal, prostate, lymphoma/leukemia, and pancreatic. The sites of cancers in the study are shown in the Supplemental Material (Table S1). About one-third of the subjects were undergoing chemotherapy.

**Table 1:** Clinical and laboratory characteristics of individuals with type 2 diabetes and cancer from the three analyses performed in the study.

	Analysis 1	Analysis 2	Analysis 3
	(Fructosamine vs. HbA1c)	(Fructosamine vs. 1-month SMBG)	(HbA1c vs. 3-month SMBG)
Variable	n = 318	n = 164	n = 111
Female sex	176 (55.3%)	72 (43.9%)	50 (45.0%)
Age (years)	65 (58‒72)	64 (57‒71)	65 (58‒73)
Fasting glucose (mg/dL)	137.0 (103.0‒190.0)	132.0 (104.0‒189.0)	125.0 (103.0‒170.0)
HbA1c (%)	8.0 (7.0‒9.4)	NA	7.6 (6.7‒8.7)
Fructosamine (µmoL/L)	315.5 (273.0‒366.8)	309.0 (268.0‒369.3)	NA
Number of BG/month	NA	63 (28)	66 (29)
Number of preprandial BG/month		48 (25)	53 (27)
Number of postprandial BG/month		15 (14)	14 (14)
1-month SMBG (mg/dL)	NA	170.1 (48.6)	NA
3-month SMBG (mg/dL)	NA	NA	168.8 (43.2)
Creatinine (mg/dL)	0.9 (0.7‒1.2)	0.9 (0.7‒1.2)	0.9 (0.7‒1.4)
eGFR (mL/min/1.73 m^2^)	81.9 (59.4‒96.3)	79.9 (58.7‒98.6)	76.2 (46.5‒94.5)
Hemoglobin (g/dL)	11.9 (11.1‒12.9)	12.2 (11.2‒13.0)	12.1 (11.1‒13.3)
Serum albumin (mg/dL)	4.2 (3.8‒4.4)	4.2 (3.9‒4.4)	4.2 (3.9‒4.4)
Serum protein (mg/dL)	6.9 (6.4‒7.3)	7.0 (6.4‒7.3)	6.9 (6.5‒7.3)
Anemia (hemoglobin <12 g/dL)	111 (45.3%)	68 (51.5%)	41 (43.6%)
Hypoalbuminemia (albumin ≤3.5 g/dL)	21 (6.6%)	10 (9.3%)	7 (9.7%)
Hypoproteinemia (total protein ≤6.5 g/dL)	54 (30.3%)	33 (30.6%)	25 (34.7%)
Cancer treatment			
Chemotherapy	96 (30.2%)	62 (37.8%)	38 (34.2%)
Glucocorticoids	101 (31.8%)	64 (39.0%)	41 (36.9%)

Data are mean (SD), median (first quartile – third quartile), or n (%). BG, Blood Glucose; eGFR, estimated Glomerular Filtration Rate; NA, Not Applicable; SD, Standard Deviation; SMBG, Self-Monitoring of Blood Glucose.

### Correlation between glycemic control measures in cancer patients with diabetes

**Fig. 1:**
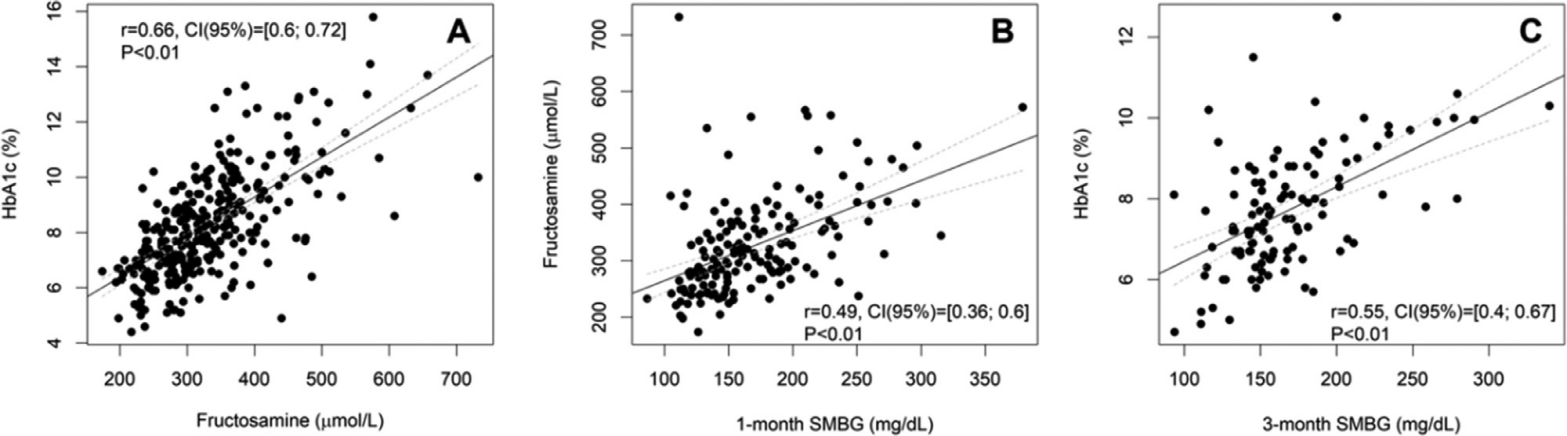
Dispersion between HbA1c and fructosamine (A), fructosamine and 1-month Self-Monitoring Blood Glucose (SMBG) (B), and HbA1c and 3-month SMBG (C) in subjects with diabetes and cancer, and the respective correlation between the variables. Gray dashed lines represent the confidence band for the regression line. The regression lines for the whole sample are [HbA1c = 0.014 × fructosamine + 3.49], [Fructosamine = 0.89 × 1-month SMBG + 176.35] and [HbA1c = 0.018 × 3-month SMBG + 4.60]. 95% CI, 95% Confidence Interval for *r. r*, Pearson's correlation coefficient.

The values of fructosamine and HbA1c of the same month for each patient had a positive and strong correlation (*r* = 0.66, 95% CI [0.60; 0.72], p < 0.001) in the analysis of data from 318 subjects. Median fructosamine was 315.5 µmoL/L (273.0‒366.8 µmoL/L) and median HbA1c was 8.0% (7.0%‒9.4%) (64 mmoL/moL [53‒79 mmoL/moL]). The dispersion between fructosamine and HbA1c measurements and the respective regression line was shown in Fig. 1A.

For the analysis between fructosamine and one-month SMBG, data from 164 subjects showed a moderate correlation between them (*r* = 0.49, 95% CI [0.36; 0.60], p < 0.001) (Fig. 1B). The median fructosamine was 309 µmoL/L (268‒369 µmoL/L) and the median 1-month SMBG was 157.2 mg/dL (135.5‒192.1).

For the analysis between HbA1c and three-month SMBG, data from 111 subjects also showed a moderate correlation between them (*r* = 0.55, 95% CI [0.40; 0.67], p < 0.001) (Fig. 1C). The median HbA1c was 7.6% (6.7%‒8.7%) (60 mmoL/moL [50‒72 mmoL/moL]) and the median 3-month SMBG was 158.1 mg/dL (143.8‒185.6).

### Comparison of glycemic control measures in cancer subjects undergoing chemotherapy or using glucocorticoids

In 101 subjects with cancer undergoing chemotherapy, the values of fructosamine and HbA1c had a positive and strong correlation (*r* = 0.61, 95% CI [0.47; 0.72], p < 0.001). For the analysis between fructosamine and 1-month SMBG, data from 64 subjects showed a strong correlation (*r* = 0.74, 95% CI [0.60; 0.83], p < 0.001). Moreover, for the analysis between HbA1c and 3-month SMBG, data from 41 subjects also showed a strong correlation (*r* = 0.67, 95% CI [0.46; 0.81], p < 0.001) (Table 2).

**Table 2:** Regression line equation variables as per subgroup analysis (chemotherapy, corticosteroids, anemia, hypoalbuminemia, hypoproteinemia, and renal function) for each correlation analysis between markers of glycemic control.

	Analysis 1					Analysis 2					Analysis 3				
	(Fructosamine vs. HbA1c)					(Fructosamine vs. 1-month SMBG)					(HbA1c vs. 3-month SMBG)				
Variable	n	*r*	a	b	p	n	*r*	a	b	p	n	*r*	a	b	p
Corticosteroid use	96	0.67	0.015	3.22	<0.001	62	0.77	1.26	95.13	<0.001	38	0.76	0.022	3.51	<0.001
On chemotherapy	101	0.61	0.015	3.55	<0.001	64	0.74	1.16	108.40	<0.001	41	0.67	0.022	3.81	<0.001
Anemia	111	0.66	0.015	3.01	<0.001	68	0.59	0.78	183.04	<0.001	41	0.75	0.020	3.94	<0.001
Hypoalbuminemia	21	0.54	0.012	4.17	0.01	10	0.77	1.08	114.24	0.01	7	0.85	0.028	2.39	0.01
Hypoproteinemia	54	0.66	0.017	3.01	<0.001	33	0.73	0.97	117.89	<0.001	25	0.80	0.027	2.76	<0.001
Normal kidney function or grade 1 and 2 CKD	189	0.70	0.015	3.35	<0.001	99	0.64	0.93	164.90	<0.001	58	0.71	0.023	3.61	<0.001
Grade 3 and 4 CKD	67	0.57	0.012	3.92	<0.001	38	0.41	0.83	198.25	0.01	34	0.44	0.014	5.36	0.01

The formula for simple linear regression is [y = ax + b], where “a” is the estimated slope, and “b” is the estimated intercept. For analysis 1, × = fructosamine and y = HbA1c; for analysis 2; × = 1-month SMBG and y = fructosamine; and for analysis 3, × = 3-month SMBG and y = HbA1c.

Abbreviations: CKD, Chronic Kidney Disease; *r*, Pearson's correlation coefficient; SMBG, Self-Monitoring of Blood Glucose.

For the analysis of the 96 cancer subjects using glucocorticoids, HbA1c, and fructosamine values were strongly correlated (*r* = 0.67, 95% CI [0.54; 0.77], p < 0.001). For the analysis between fructosamine and 1-month SMBG, data from 62 subjects showed a strong correlation (*r* = 0.77, 95% CI [0.64; 0.85], p < 0.001). Furthermore, for the analysis between HbA1c and 3-month SMBG, data from 38 subjects also showed a strong correlation (*r* = 0.76, 95% CI [0.58; 0.87], p < 0.001) (Table 2).

### Comparison of glycemic control measures in cancer subjects with anemia, hypoalbuminemia, hypoproteinemia and according to renal function

In 111 subjects with anemia, fructosamine, and HbA1c values were strongly correlated (*r* = 0.66, 95% CI [0.54; 0.76], p < 0.001). For the analysis between fructosamine and 1-month SMBG, data from 68 subjects with anemia showed a moderate correlation (*r* = 0.54, 95% CI [0.34; 0.69], p < 0.001). In addition, for the analysis between HbA1c and 3-month SMBG, data from 41 subjects with anemia showed a strong correlation (*r* = 0.75, 95% CI [0.57; 0.86], p < 0.001) (Table 2).

Fructosamine and HbA1c values were moderately correlated in 21 subjects with hypoalbuminemia (*r* = 0.54, 95% CI [0.14; 0.79], p *=* 0.001) and strongly correlated in 54 subjects with hypoproteinemia (*r* = 0.66, 95% CI [0.48; 0.79], R^2^ = 0.44, p < 0.001). Fructosamine and 1-month SMBG were strongly correlated in 33 subjects with hypoproteinemia (*r* = 0.73, 95% CI [0.51; 0.86], p < 0.001) and in 10 subjects with hypoalbuminemia (*r* = 0.77, 95% CI [0.27; 0.94], p = 0.01). HbA1c and 3-month SMBG were strongly correlated in seven subjects with hypoalbuminemia (*r* = 0.85, 95% CI [0.26; 0.98], p = 0.02) and in 25 subjects with hypoproteinemia (*r* = 0.80, 95% CI [0.60; 0.91], p < 0.001) (Table 2).

In subjects with normal kidney function or grade 1 and 2 CKD (eGFR ≥ 60 mL/min/1.73 m^2^) fructosamine and HbA1c values were strongly correlated (n=189, *r* = 0.70, 95% CI [0.62; 0.77], p < 0.001); fructosamine and 1-month SMBG, moderately correlated (n = 99, *r* = 0.51, 95% CI [0.34; 0.64], p < 0.001); and HbA1c and three-month SMBG, strongly correlated (n = 58, *r* = 0.71, 95% CI [0.55; 0.82], p < 0.001). In subjects with grade 3 and 4 CKD (eGFR < 60 mL/min/1.73 m^2^) the three correlation analyses showed a moderate correlation (fructosamine and HbA1c: n = 67, *r* = 0.57, 95% CI [0.38; 0.71], p < 0.001; fructosamine and 1-month SMBG: n = 38, *r* = 0.41, 95% CI [0.10; 0.64], p = 0.001; HbA1c and 3-month SMBG: n = 34, *r* = 0.44, 95% CI 0.12; 0.67], p = 0.01) (Table 2).

The components of the regression line equations, according to subgroup analysis (chemotherapy, glucocorticoids, anemia, hypoalbuminemia, hypoproteinemia, and renal function) are available in Table 2.

## Discussion

The present study evaluated whether serum fructosamine and HbA1c may be used to assess short-term and long-term glycemic control, respectively, in subjects with diabetes and cancer, including those undergoing chemotherapy. Cancer patients have several particularities, such as chemotherapy treatment, the use of corticosteroids, the presence of anemia, cachexia, or malnutrition, which can affect glycemic biomarkers.^11^^,^^20^

The HbA1c test is an indirect measure of average blood glucose over the past 90‒120 days.^6^^,^^21^ Conditions that affect erythrocyte turnover (anemia, hemolysis, drugs that stimulate erythropoiesis, end-stage renal disease, and pregnancy), recent blood transfusion, and hemoglobin variants can cause discrepancies between the HbA1c and the mean SMBG over the last two to three months.^3^^,^^5^, ^6^, ^7^ Other tests such as serum fructosamine and urinary 1,5-anhydroglucitol are available as markers of short-term glycemic control.^3^^,^^5^^,^^7^

One study evaluated the correlation between HbA1c and fructosamine in 153 subjects with diabetes, whose regression line was [HbA1c = 0.017 × fructosamine + 1.61] (*r* = 0.78).^22^ Another study evaluated the correlation between HbA1c and fructosamine in individuals with diagnosed type 2 diabetes previously, newly diagnosed type 2 diabetes, and type 1 diabetes, also showing a good correlation. In this study, the repeated measures subanalysis of fructosamine and HbA1c showed that the correlation coefficients were similar regardless of the follow-up time.^7^ The present study showed a good correlation between HbA1c and fructosamine and enabled the construction of an equation derived from the regression line specifically for individuals with cancer: (HbA1c = 0.014 × fructosamine + 3.49). This study also showed a good correlation between fructosamine and HbA1c in subjects with diabetes and cancer, even in those who underwent chemotherapy or corticosteroid use, and those with anemia, hypoalbuminemia, hypoproteinemia, or eGFR less than 60 mL/min/1.73 m^2^.

Fructosamine is a ketoamine formed by the non-enzymatic glycation of the amino group of proteins, mostly albumin.^7^^,^^21^^,^^23^, ^24^, ^25^, ^26^ Fructosamine assays are cheap, robust, and easy to perform^7^^,^^8^^,^^10^ and their results reflect average blood glucose concentrations over the previous two to three weeks, which can be used clinically as markers of recent changes in glycemic control.^7^^,^^10^^,^^25^^,^^26^ Furthermore, fructosamine can be reliably measured regardless of fasting or non-fasting.^7^ To assess whether fructosamine reflects one-month SMBG, the correlation between these variables was assessed. In this present study, there was a good correlation between serum fructosamine and 1-month mean SMBG in subjects with cancer, including those on chemotherapy or on glucocorticoids. Fructosamine showed a strong correlation with both HbA1c and 1-month SMBG in those with hypoproteinemia. In a cancer patient, several factors can affect serum albumin and total protein concentrations, such as malnutrition, cachexia, tumor necrosis, chemotherapy-induced hepatotoxicity, and inflammatory states,^27^^,^^28^ making the understanding of protein metabolism more challenging.

Anemia is present in 30% to 90% of cancer subjects.^29^ Therefore, such variation in the blood count may affect the value of HbA1c during chemotherapy. Many chemotherapy drugs induce myelotoxicity, resulting in suppression of hematopoiesis.^30^ In addition, many subjects may need a transfusion of blood components during cancer follow-up. In the present study, the prevalence of anemia was around 45% and the authors excluded data from subjects who received blood transfusions. HbA1c showed good correlations with three-month SMBG. Correlations were also good in individuals undergoing chemotherapy, using glucocorticoids, and even those with anemia.

This study did not include subjects with stage 5 CKD, but the present results showed that fructosamine and HbA1c correlated well with SMBG, even in subjects with stage 3 and 4 CKD, where kidney function is already more deteriorated, and anemia is a prominent clinical feature.

Fructosamine and HbA1c are complementary to SMBG and not substitutes. However, in a patient with good glycemic control, these tests can be useful tools to monitor possible changes in glycemic control, even helping to assess glycemic control despite the difficulty of an oncology patient in performing SMBG. Physicians treating subjects with diabetes should be familiar with a broader definition of optimal glycemic control, drawing on an arsenal of metrics that more realistically reflect the dynamic nature of glycemic control^31^, especially in the patient undergoing cancer treatment.

This study had some limitations, including those inherent in a retrospective study of a single institution. Despite the recommendation to perform at least two to three BG measurements per day, the number of measurements varied considerably. Blood glucose test strips were not provided by the present study's hospital. Thus, capillary blood glucose data were more difficult to obtain for three consecutive months, especially outside the chemotherapy period, for the comparative analysis with HbA1c and, consequently, the number of subjects for this analysis was smaller. The types of cancer and chemotherapy treatment were evaluated in a generalized way, especially considering the tumor topography. The standard deviation of the hemoglobin values was low, indicating that participants with hemoglobin values <12 g/dL had mild anemia in this study. Specific studies for individuals with more severe anemia are needed. The myelotoxicity potential of chemotherapy and the use of erythropoietin, a medication frequently used in cancer subjects, have not been evaluated. However, subjects with more severe renal impairment (stage 5 CKD) were excluded from the study. Specific studies for each neoplasm and specific chemotherapy would be of great value for a more detailed understanding of HbA1c and fructosamine's role in these populations. The authors were careful to exclude subjects with hematologic malignancies involving paraproteins from analyses involving fructosamine (Supplemental Material – Table S1). Individuals with type 1 diabetes mellitus were excluded from the analysis due to the small number of cases.

## Conclusions

In conclusion, there is a strong positive correlation between fructosamine and HbA1c in people with diabetes and cancer, even those on chemotherapy, using glucocorticoids, and with anemia. Both HbA1c and fructosamine had at least a moderate correlation with SMBG, being stronger in subjects undergoing chemotherapy. These results support the use of glycated hemoglobin and fructosamine as glycemic biomarkers in patients with diabetes and cancer, including those undergoing chemotherapy, with anemia or hypoproteinemia.

## Authors’ contributions

M.T.K.T. wrote the initial research proposal and manuscript. M.T.K.T, P.C., E.Z.K. and C.A.M. collected and researched the data. A.S.D. and R.L.B. contributed to statistical analysis. M.T.K.T., P.C., A.S.D., R.L.B., F.A.C., C.A.M., A.O.H. and M.N. reviewed/edited the research proposal and manuscript and contributed to the discussion.

## Data availability

Original data generated and analyzed during this study are included in this published article. Other data are available from the corresponding author upon reasonable request.

## Funding

This research did not receive any specific grant from funding agencies in the public, commercial, or not-for-profit sectors.

## Conflicts of interest

There authors declare no conflicts of interest. All authors have read and approved the submission of the manuscript; the manuscript has not been published and is not being considered for publication elsewhere, in whole or in part, in any language.

## Appendix Supplementary materials

Image, application 1
