# Supplementary material for: Fructosamine and glycated hemoglobin as biomarkers of glycemic control in people with type 2 diabetes mellitus and cancer (GlicoOnco study)
Source: Clinics (Sao Paulo). 2023 Jun 28;78:100240. doi: 10.1016/j.clinsp.2023.100240 (PMC10338289; doi:10.1016/j.clinsp.2023.100240)
Supplement: Supplementary file 1 [file mmc1.docx]

CLINICS-D-23-00182_Supplementary Material

**Table S1** Cancer sites of individuals with diabetes and cancer from the three analyses performed in the study.

|  | **Analysis 1** | **Analysis 2** | **Analysis 3** |
| --- | --- | --- | --- |
|  | **(Fructosamine vs. HbA1c)** | **(Fructosamine vs. 1-month SMBG)** | **(HbA1c vs. 3-month SMBG)** |
| **Variable** | **n = 318** | **n = 164** | **n = 111** |
| **Cancer sites** |  |  |  |
| **Breast** | 54 (17.0%) | 20 (12.2%) | 15 (13.5%) |
| **Colorectal** | 47 (14.8%) | 28 (17.1%) | 17 (15.3%) |
| **Prostate** | 32 (10.1%) | 17 (7.0%) | 10 (6.9%) |
| **Lymphoma/Leukemia** | 32 (10.1%) | 12 (7.3%) | 7 (6.3%) |
| **Paraprotein-assocated** | 0 (0.0%) | 0 (0.0%) | 10 (9.0%) |
| **Pancreatic** | 21 (6.6%) | 16 (9.8%) | 13 (11.7%) |
| **Neuroendocrine** | 14 (4.4%) | 6 (3.7%) | 4 (3.6%) |
| **Thyroid** | 14 (4.4%) | 7 (4.3%) | 3 (2.7%) |
| **Uterine** | 14 (4.4%) | 9 (5.5%) | 5 (4.5%) |
| **Liver** | 12 (3.8%) | 5 (3.0%) | 3 (2.7%) |
| **Ovarian** | 10 (3.1%) | 4 (2.4%) | 2 (1.8%) |
| **Bladder** | 8 (2.5%) | 3 (1.8%) | 2 (1.8%) |
| **Sarcoma** | 8 (2.5%) | 6 (3.7%) | 4 (3.6%) |
| **Central nervous system** | 7 (2.2%) | 5 (3.0%) | 3 (2.7%) |
| **Melanoma** | 7 (2.2%) | 3 (1.8%) | 0 (0.0%) |
| **Neoplastic syndromes** | 7 (2.2%) | 4 (2.4%) | 2 (1.8%) |
| **Lung** | 7 (2.2%) | 3 (1.8%) | 3 (2.7%) |
| **Gastroduodenal** | 6 (1.9%) | 6 (3.7%) | 4 (3.6%) |
| **Head and Neck** | 6 (1.9%) | 3 (1.8%) | 1 (0.9%) |
| **Kidney** | 5 (1.6%) | 4 (2.4%) | 2 (1.8%) |
| **Esophageal** | 4 (1.3%) | 2 (1.2%) | 2 (1.8%) |
| **Non-melanoma skin** | 3 (0.9%) | 1 (0.6%) | 0 (0.0%) |

Data are n (%). SMBG, Self-Monitoring of Blood Glucose.
